# Supplementary material for: From sequence to enzyme mechanism using multi-label machine learning
Source: BMC Bioinformatics. 2014 May 19;15:150. doi: 10.1186/1471-2105-15-150 (PMC4229970; doi:10.1186/1471-2105-15-150)
Supplement: Additional file 2 — Java code of ml2db. Additional file ml2db_code.tar.gz contains the Java source code to run the multi-label machine learning experiments and save the results to database. The code’s Javadoc is included. [file 1471-2105-15-150-S2.zip › additional file 2/ml2db/utils/doc/uk/ac/standrews/utils/main/database/TableColumn.html]

TableColumn


JavaScript is disabled on your browser.


- Overview
- Package
- Class
- Use
- Tree
- Deprecated
- Index
- Help

- Prev Class
- Next Class

- Frames
- No Frames

- All Classes

- Summary:
- Nested |
- Field |
- Constr |
- Method

- Detail:
- Field |
- Constr |
- Method


uk.ac.standrews.utils.main.database

## Class TableColumn

- java.lang.Object
- - uk.ac.standrews.utils.main.database.TableColumn

- All Implemented Interfaces:
  :   java.lang.Comparable<TableColumn>

  ---

    

  ```
  public class TableColumn
  extends java.lang.Object
  implements java.lang.Comparable<TableColumn>
  ```

  A column object contains all the information to create, fill and delete a
  database table column

  Version:
  :   24-Mar-2007

  Author:
  :   Luna - luna.deferrari@ed.ac.uk

- - ### Constructor Summary

    Constructors

    Constructor and Description

    `TableColumn(java.lang.String columnName,
    java.lang.String dataType,
    boolean isKey)`

    Constructor 2: takes the column name, data type and primary key status
  - ### Method Summary

    Methods

    | Modifier and Type | Method and Description |
    | --- | --- |
    | `int` | `compare(TableColumn f1, TableColumn f2)` Compares two columns by their name, returns -1 if f1 comes alphabetically before f2, 0 if the two are equal and +1 if f2 comes alphabetically before f1. |
    | `int` | `compareTo(TableColumn f2)` Compares two columns by their name, returns -1 if this column comes alphabetically before f2, 0 if the two are equal and +1 if f2 comes alphabetically before this column. |
    | `boolean` | `equals(TableColumn anotherColumn)` Compares two columns by their name, returns true if the two columns have the same name, false otherwise. |
    | `java.lang.String` | `getColumnName()` Gets the column name |
    | `java.lang.String` | `getDataType()` Gets the column data type |
    | `int` | `getNoOfRows()` Gets the number of rows in the column |
    | `boolean` | `isPrimaryKey()` Gets the column primary key status |
    | `void` | `setIsPrimaryKey(boolean isKey)` Sets the column primary key status |

    - ### Methods inherited from class java.lang.Object

      `equals, getClass, hashCode, notify, notifyAll, toString, wait, wait, wait`

- - ### Constructor Detail


    - #### TableColumn

      ```
      public TableColumn(java.lang.String columnName,
                 java.lang.String dataType,
                 boolean isKey)
      ```

      Constructor 2: takes the column name, data type and primary key status

      Parameters:
      :   `columnName` - name of the column
      :   `dataType` - data type (e.g. varchar(25) )
      :   `isKey` - true if the column is a primary key
  - ### Method Detail


    - #### compare

      ```
      public int compare(TableColumn f1,
                TableColumn f2)
      ```

      Compares two columns by their name, returns -1 if f1 comes alphabetically
      before f2, 0 if the two are equal and +1 if f2 comes alphabetically
      before f1.

      Parameters:
      :   `f1` - the first column to compare
      :   `f2` - the second column to compare

      Returns:
      :   returns -1 if this column is smaller than f2, 0 if equal and +1
          if greater (in alphabetical order)


    - #### compareTo

      ```
      public int compareTo(TableColumn f2)
      ```

      Compares two columns by their name, returns -1 if this column comes
      alphabetically before f2, 0 if the two are equal and +1 if f2 comes
      alphabetically before this column.

      **Specified by:**
      :   `compareTo` in interface `java.lang.Comparable<TableColumn>`

      Parameters:
      :   `f2` - the second column to compare

      Returns:
      :   returns -1 if this column is smaller than f2, 0 if equal and +1
          if greater (in alphabetical order)


    - #### equals

      ```
      public boolean equals(TableColumn anotherColumn)
      ```

      Compares two columns by their name, returns true if the two columns have
      the same name, false otherwise.

      Parameters:
      :   `anotherColumn` - the second column to compare

      Returns:
      :   returns true if the two columns have the same name, false
          otherwise.


    - #### getColumnName

      ```
      public java.lang.String getColumnName()
      ```

      Gets the column name

      Returns:
      :   the column name


    - #### getDataType

      ```
      public java.lang.String getDataType()
      ```

      Gets the column data type

      Returns:
      :   the column data type, e.g. VARCHAR(10)


    - #### getNoOfRows

      ```
      public int getNoOfRows()
      ```

      Gets the number of rows in the column

      Returns:
      :   the number of rows in the column


    - #### isPrimaryKey

      ```
      public boolean isPrimaryKey()
      ```

      Gets the column primary key status

      Returns:
      :   the primary key status, e.g. 'true'= is a primary key


    - #### setIsPrimaryKey

      ```
      public void setIsPrimaryKey(boolean isKey)
      ```

      Sets the column primary key status

      Parameters:
      :   `isKey` - the primary key status, e.g. 'true'= is a primary key


- Overview
- Package
- Class
- Use
- Tree
- Deprecated
- Index
- Help

- Prev Class
- Next Class

- Frames
- No Frames

- All Classes

- Summary:
- Nested |
- Field |
- Constr |
- Method

- Detail:
- Field |
- Constr |
- Method
